# Applied phyloepidemiology: Detecting drivers of pathogen transmission from genomic signatures using density measures

**DOI:** 10.1111/eva.12991

**Published:** 2020-05-22

**Authors:** Thierry Wirth, Vanessa Wong, François Vandenesch, Jean‐Philippe Rasigade

**Affiliations:** ^1^ Institut de Systématique, Evolution, Biodiversité UMR‐CNRS 7205 Muséum National d’Histoire Naturelle Université Pierre et Marie Curie Université des Antilles Ecole Pratique des Hautes Etudes Sorbonne Universités Paris France; ^2^ EPHE PSL University Paris France; ^3^ Cambridge Institute for Medical Research Welcome Trust Center Cambridge UK; ^4^ CIRI INSERM U1111 CNRS UMR5308 ENS Lyon University of Lyon Lyon France; ^5^ Institut des Agents Infectieux Hospices Civils de Lyon Lyon France

**Keywords:** epidemic, fitness cost, fluoroquinolone resistance, *Salmonella*, *Staphylococcus*, THD, tuberculosis

## Abstract

Understanding the driving forces of an epidemic is key to inform intervention strategies against it. Correlating measures of the epidemic success of a pathogen with ancillary parameters such as its drug resistance profile provides a flexible tool to identify such driving forces. The recently described time‐scaled haplotypic density (THD) method facilitates the inference of a pathogen's epidemic success from genetic data. Contrary to demogenetic approaches that define success in an aggregated fashion, the THD computes an independent index of success for each isolate in a collection. Modeling this index using multivariate regression, thus, allows us to control for various sources of bias and to identify independent predictors of success. We illustrate the use of THD to address key questions regarding three exemplary epidemics of multidrug‐resistant (MDR) bacterial lineages, namely *Mycobacterium tuberculosis* Beijing, *Salmonella* Typhi H58, and *Staphylococcus aureus* ST8 (including ST8‐USA300 MRSA), based on previously published, international genetic datasets. In each case, THD analysis allowed to identify the impact, or lack thereof, of various factors on the epidemic success, independent of confounding by population structure and geographic distribution. Our results suggest that rifampicin resistance drives the MDR Beijing epidemic and that fluoroquinolone resistance drives the *S. aureus* ST8/USA300 epidemic, in line with previous evidence of a lack of resistance‐associated fitness cost in these pathogens. Conversely, fluoroquinolone resistance measurably hampered the success of *S. *Typhi H58 and non‐H58. These findings illustrate how THD can help leverage the massive genomic datasets generated by molecular epidemiology studies to address new questions. THD implementation for the R platform is available at https://github.com/rasigadelab/thd.

## 1 INTRODUCTION

In a globalizing world where re‐emerging diseases and antibiotic‐resistant pathogens threaten the health systems of numerous countries (Wirth, 2018), there is an urgent need for simple and efficient tools to quantify pathogen transmission. Correlating this information with ancillary parameters such as antibiotic resistance profiles, pathogen demogenetic measures or host characteristics (e.g., vaccine status) can then help to determine the driving forces behind the epidemic success of a pathogen. Finally, understanding these driving forces allows us to identify modifiable drivers of an epidemic and to inform intervention strategies against it (Heesterbeek et al., 2015).

The concepts of epidemicity and endemicity are central to infectious diseases epidemiology. Endemic areas for an infection are where the microbe and its related clinical cases persist over years, displaying a continuous incidence. The continuity rather than the intensity of the incidence is the effective indicator of endemicity. This concept was illustrated by the calculation of an index of endemicity relying on the study of cholera mortality over long periods of time in Bengal (Kamal, 1963). Contrasting with endemicity, epidemicity characterizes the dynamics of the infection. Epidemicity can reflect, for instance, the spread of a pathogen in an empty or naïve niche with a sudden burst followed by an abrupt ending, as exemplified by the *Vibrio cholerae* outbreak in Haiti in 2010 (Eppinger et al., 2014), or a novel zoonosis like HIV in the 1980s (Faria et al., 2014). Other situations are less clear‐cut, such as when local outbreaks occur in otherwise endemic areas. When linked with environmental factors, such outbreaks can appear as seasonal or completely stochastic. Importantly, epidemicity is tightly linked to the concepts of epidemic success and fitness. In the following, we use the term epidemicity to qualify the spread of a disease in an epidemiological context, while the term epidemic success relates to the success of the causative pathogen from an evolutionary standpoint. Finally, the fitness of the pathogen qualifies the biological characteristic that underlies its epidemic success in specific conditions. These distinctions are meant to emphasize the chain of consequences in which a pathogen's fitness enhances its epidemic success and, in turn, the epidemicity of the disease.

Our understanding of the dynamics of infectious disease epidemics has been facilitated by the continued improvement of modeling techniques (Cauchemez, Hoze, Cousien, Nikolay, & Ten Bosch, 2019), based on two major paradigms: (a) epidemiological models (Anderson & May, 1992; Keeling & Rohani, 2008) that consider the dynamics of an entire population going forward in time and (b) coalescent theory (Kingman, 1982), which considers small samples of an infected population and operates backward in time until the common ancestor has been reached. Although historical data on transmission dynamics are frequently lacking, the genotypes of extant pathogens are increasingly available. Therefore, using these genetic data to infer the epidemic success of a pathogen or group of pathogens can greatly improve our understanding of the drivers of pathogen transmission and spread.

Here, we illustrate applications of the time‐scaled haplotypic density (THD), a recently developed measure of epidemic success (Barbier et al., 2018; Rasigade et al., 2017), to model the driving forces behind major pathogen epidemics, using rich genetic datasets on three exemplary human pathogens. After discussing the motivation for the THD method and its mathematical foundations, we first revisit the *Mycobacterium tuberculosis* Beijing epidemic in Eurasia (Merker et al., 2015) and establish links between patterns of antimicrobial resistance and epidemic success. In a second example, we apply THD to infer whether fluoroquinolone resistance impacts fitness in *Salmonella* Typhi populations, including the pandemic, multidrug‐resistant H58 lineage (Wong et al., 2015). Finally, we examine whether the epidemic success of distinct lineages of community‐acquired methicillin‐resistant *Staphylococcus aureus* was measurably influenced by two previously suspected success‐associated traits, namely fluoroquinolone resistance and the arginine catabolic mobile element ACME (Glaser et al., 2016).

## 2 MOTIVATION FOR AN INDIVIDUAL‐BASED CORRELATE OF EPIDEMIC SUCCESS

In the following section, we relate the epidemic success of a pathogen to the density of transmission events in its ancestry. If a pathogen's evolution rate is fast enough, each transmission event results in a genetic divergence event that can be inferred as a node in the phylogeny reconstructed from the genotypes of the descendants of the transmitted pathogen. Based on this assumption, the phylogenetic signature of epidemic success is the density of divergence events in the pathogen's ancestry.

Several demogenetic and phyloepidemiology techniques estimate quantities that reflect the density of divergence events in the ancestry of a population. For instance, the expansion rate of a pathogen population *r*, or Malthusian parameter, can be estimated under the exponential growth model *N*(*t*) = *e^rt^* *N_0_* where *N*(*t*) is the population size after *t* epochs and *N*_0_ is the initial population size. More specialized phylodynamics models have been adapted to the epidemic setting to explicitly consider host infection and recovery and the detection probability of a pathogen (Popinga, Vaughan, Stadler, & Drummond, 2015; Volz, Kosakovsky Pond, Ward, Leigh Brown, & Frost, 2009). Such models can infer the basic reproduction number *R*_0_ of an epidemic, which is the number of secondary cases of infection generated by an index case during the infectious period.

Importantly, correlates of epidemic success such as the Malthusian parameter or the basic reproduction number are defined at the level of a group of pathogens, that is, a population. These correlates are useful for comparing the epidemic success of distinct pathogen species or lineages. Because of their aggregated nature, however, such correlates of epidemic success cannot be used to infer conclusions at the individual level, as such conclusions can be contaminated by cross‐sectional effects (Schechner, Temkin, Harbarth, Carmeli, & Schwaber, 2013). Correlation between group‐level features, such as between a correlate of epidemic success and other pathogen characteristics, does not imply correlation at the individual level because between‐group correlation can result from correlation between the features in distinct individuals of the same group.

These limitations of group‐level correlates of epidemic success in correlation analysis can be circumvented by constructing models that directly relate pathogen characteristics with the rate of divergence events in a phylogenetic tree. Popular models that build upon this idea belong to the category of state‐dependent diversification models, such as the binary‐state speciation and extinction (BiSSE) model (Maddison, Midford, & Otto, 2007) or the Bayesian analysis of macroevolutionary mixtures (BAMM) (Rabosky, 2014), whose validity was criticized by several authors (Rabosky, Mitchell, & Chang, 2017). Because of their sophistication and computational demand, however, state‐dependent diversification models can be difficult to use with large datasets (typically, *n* > 1,000) and the multiple‐feature hypotheses that are increasingly common in pathogen epidemic studies.

To circumvent the statistical drawbacks of group‐based correlates of epidemic success and the complexity of state‐dependent diversification models, our group proposed a simpler, alternative approach to epidemicity analysis that provides individual‐based correlates. The expected benefit of defining correlates of epidemic success at the individual level is to facilitate the detection of factors associated with epidemic success using regression models that can control for multiple confounders (Box 1).

BOX 1 **Applied relevance**

Fitness is a key concept in the field of evolutionary biology and describes how good a genotype is at spreading its genes to the next generations. This handy concept is however more difficult to grasp in the field of microbiology. Microbial pathogens’ success is often associated with a higher epidemicity of the corresponding disease, but pertinent individual‐based measures of epidemic success are still lacking. Here, we illustrate the application of a recently developed measure of epidemic success, the time‐scaled haplotypic density (THD), to model the driving forces behind major epidemics of multidrug‐resistant bacterial pathogens.

The THD index has the potential to be implemented in genome wide association studies (GWAS). Treated as a quantitative trait, THD could identify SNPs or more complex genetic architectures associated with epidemic success. The widespread adoption of THD‐based GWAS might help expand the spectrum of the candidate genes or mutations linked to pathogen success, thus accompanying a shift from the current, paradigmatic antibiotic resistance‐targeted GWAS approaches to more general questions pertaining to pathogen ecology and evolution. From a more applied point of view, the THD might allow us to pinpoint SNPs of major importance linked to virulence and spread in pathogens as well as to extend our knowledge concerning the adaptive landscape of those germs.

## 3 RELATIONSHIPS BETWEEN EPIDEMIC SUCCESS AND DENSITY IN THE SPACE OF GENETIC DISTANCES

All else being equal, the epidemic success in a group of pathogens increases its prevalence faster than its diversity, resulting in a more uniform (i.e., less diverse) genetic population structure compared to other groups in the sample. Lower diversity results in smaller genetic distances between isolates. From a statistical standpoint, both the prevalence of and pairwise genetic distances between isolates in a group can be jointly quantified by a measure of density in the space of genetic distances, suggesting that density correlates with success. Contrary to the aggregated measures discussed above, density is defined at all points in the space of genetic distances, hence on the level of individuals in the population.

Time‐scaled haplotypic density defines the density measure using an application‐specific adaptation of kernel density estimation (KDE) (Parzen, 1962). In the general case, KDE computes density based on distances between points and a kernel function with a bandwidth parameter to control the smoothness of the estimate. In the THD setting, points are haplotypes represented as vectors of markers, distances are the pairwise numbers of allelic differences and the kernel function is based on the geometric distribution. To control the bandwidth of the analysis in an interpretable fashion, the THD bandwidth is expressed in units of time rather than genetic distance.

The THD computation proceeds as follows. Let *X* be a sample of *n* haplotypes defined over *m* markers, represented as an (*n* × *m*) data matrix, and let *y* be a haplotype of interest not in *X*, for which the density is to be computed. For each haplotype *x_i_* in *X*, let *h_i_* be the Hamming genetic distance from *y* to *x_i_*, that is, the number of differences between *x_i_* and *y*. A distance *h* is associated with a kernel density (formally, a probability) under the truncated geometric distribution with bandwidth *b* (formally, the failure probability of a Bernoulli trial) and truncation limit *m*. This distribution has probability mass function $k\left(h|b,m\right)=\left(\frac{1-b}{1-b^{m+1}}\right)b^{h}$ where the bandwidth *b* is a real number in [0, 1). Remark that the density is proportional to *b^h^*, which illustrates how the bandwidth controls the influence of the distance *h* on the density: For each additional difference between *y* and *x*, the density is multiplied by *b*. Reducing *b*, thus, accelerates the decrease of the density for larger numbers of differences. Finally, the haplotypic density *K* (*y* | *X*, *b*, *m*) of *y* with respect to *X* is the average of the *n* densities associated with the distances from *y* to each *x_i_* in *X*,

$$K\left(y|X,b,m\right)=\frac{1}{n}\sum_{i}^{n}k\left(h_{i}|b,m\right)=\frac{1}{n}\left(\frac{1-b}{1-b^{m+1}}\right)\sum_{i}^{n}b^{h_{i}}.$$

Because *b* is a dimensionless constant, its choice is not intuitive. To circumvent this issue, we exploit the existence of a bijective relationship between the genetic distance *h* and the maximum‐likelihood estimate *t* of the time to the most recent common ancestor (TMRCA) under the infinite alleles model (IAM) (Kimura & Crow, 1964; Walsh, 2001). The IAM assumes that the *m* haplotype markers lie on a non‐recombining DNA segment, that they evolve independently with a common evolutionary rate *µ*, and that at most one change per marker has occurred in both lineages since their MRCA. Assuming that *µ* is known, the IAM allows us to replace the bandwidth with a more intuitive timescale parameter *t*_50_, which is the TMRCA such that haplotypes with shorter TMRCAs account for 50% of the density. Practically, we solve the IAM relation *t* = log[*m*/(*m* − *h*)]/2*μ* for *h* to obtain *h* = (1 − *e* ^‐2^ *^μt^*)*m*. This relation associates a distance *h*_50_ with the chosen timescale *t*_50_. From the definition of *t*_50_, it follows that *h*_50_ is the median of a truncated geometric distribution whose bandwith b_*_ must be determined. From the cumulative probability function of the truncated geometric distribution with parameters *b* and *m*, $P\left(H\leq h|b,m\right)=\frac{1-b^{h}}{1-b^{m}},$ it follows that if *h*_50_ is the median of the continuous form of the distribution with bandwidth b_*_ then b_*_ must satisfy $\frac{1-b_{*}^{h_{50}}}{1-b_{*}^{m}}=\frac{1}{2}$ . While this last equation has no analytic solution, it is easily solved for b_*_ numerically using a root‐finding algorithm over the [0, 1) interval.

From a computational complexity standpoint, the THD method scales linearly with the size of the input matrix of genetic distances, hence quadratically with the sample size. The single‐threaded computation time is ~0.1 s for a sample size of 1,000 isolates on a standard computer (Intel I7 CPU at 3.4 GHz). As a comparison point, this computation is two orders of magnitude faster than a typical, fast phylogeny reconstruction method (BIONJ) applied on the same dataset (Figure S1).

**FIGURE 1:**
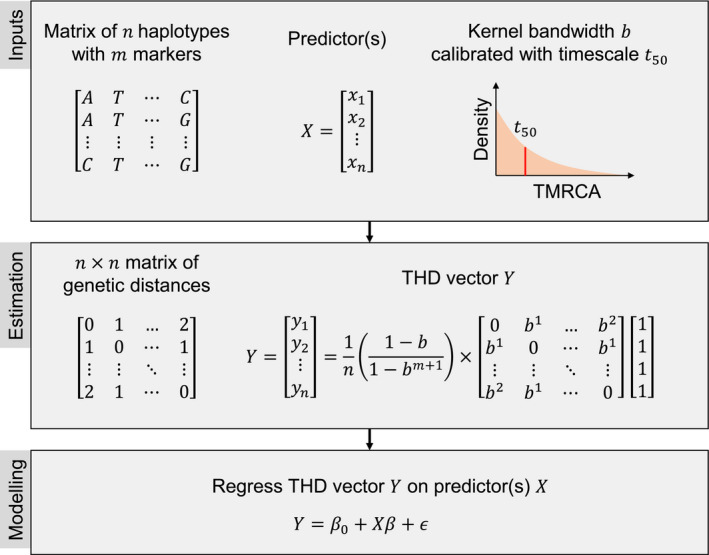
Overview of the THD method's inputs, estimation, and modeling steps. THD estimation is a special case of kernel density estimation where the observations are haplotypes and the kernel bandwidth *b* is calibrated relative to a timescale parameter *t*_50_ defined as the median of the kernel distribution. The explicit estimation formula emphasizes that THD estimation has *O* (*n* ^2^) space and time complexity. THD values, used as proxies for the epidemic success of each haplotype, are typically regressed on a set of predictor variables. TMRCA, time to the most recent common ancestor between two haplotypes. *ϵ* is the error term of the regression model

The THD computation steps can be summarized as follows: (a) determine parameters *m* (number of markers), *µ* (evolutionary rate) and *t*_50_ (timescale); (b) associate the timescale with a median distance *h*_50_; (c) determine the corresponding bandwidth b_*_; and (d) for each haplotype of interest, compute THD as the average kernel density under the truncated geometric distribution with bandwidth b_*_ and truncation limit *m*. The resulting vector of THD values is typically used as the response variable of linear regression models using potential predictors of epidemic success as the explanatory variables. A summary of the model estimation steps is shown in Figure 1. Practical guidelines on parameter selection and model interpretation are proposed in Box 2. A reference implementation of THD for the R platform is available at https://github.com/rasigadelab/thd.

BOX 2 **Guidelines for THD modeling and interpretation**

THD modeling seeks to examine associations between the epidemic success and the characteristics of a pathogen or its host. We propose a synthetic overview of the construction and interpretation of linear regression models that use THD is as the response variable.

**Selection of explanatory variables**

Potential predictors of epidemic success can relate to the host, such as the sociodemographic or clinical characteristics of infected patients (Rasigade et al., 2017), or to the pathogen, such as its phenotype or genotype, including single nucleotide polymorphisms. Special care should be taken to identify potential confounders to be included as model covariates.

**Timescale selection**

The timescale *t*_50_ controls how fast the contribution of isolates to the density decreases with the age of their divergence (the time to the most recent common ancestor, TMRCA). Technically, *t*_50_ is the TMRCA such that isolates with a TMRCA at most equal to *t*_50_ account for 50% of the density. Practically, the timescale reflects the time window of interest depending on the study question. A sensible choice for *t*_50_ is the time elapsed since the onset of the pathogen spread. Shorter timescales can be used to model smaller‐scale phenomena such as the influence of host‐related predictors that do not depend on pathogen evolution.

**Controlling for population structure**

THD values exhibit a strong phylogenetic autocorrelation (that is, closely related isolates tend to have similar THDs) because THD depends by design on the population structure. Comparing THD models with and without controlling for population structure provides information on the relationship of the predictor under study with epidemic success. In THD models, population structure is treated as a potential confounder of this relationship.

A predictor‐THD association that remains significant in the controlled model is independent of population structure, that is, the association is not explained by a shared evolutionary history of the predictor and the pathogen's fitness. Population structure‐independent associations typically arise when: (a) the predictor characterizes the pathogen's host or environment rather than the pathogen itself; and (b) the predictor is a pathogen trait that evolved repeatedly in different lineages or within them. This latter case indicates convergent evolution, which supports a causal role of the predictor in increasing the epidemic success.

If the predictor–THD association is significant in the uncontrolled model but not in the controlled model, the predictor is said to be conditionally independent of THD given population structure. A population structure‐dependent association arises for instance when the predictor evolved in a unique lineage whose epidemic success differs from other lineages. In such a case, the predictor might or might not play a causal role because its evolutionary history is intertwinned with that of the variation of epidemic success. In other words, the predictor can be considered a marker of the successful lineage but not necessarily the cause of this success.

**Practical methods for population structure control**

Controlling for population structure can be achieved using several methods (reviewed in Sul, Martin, & Eskin, 2018). We focus on the simplest ones, namely random‐effects grouping and principal coordinates analysis. Random‐effects grouping can be applied when the population structure can be described by grouping isolates in several well‐defined clades, such as major lineages or clonal complexes. Random‐effects models use between‐group variance internally; hence, a sufficient number of groups (typically >5) must be present in the sample for the variance estimate to be reliable. If isolates cannot be grouped in a meaningful fashion, population structure can be accounted for by introducing genetic principal coordinates (PCs) as model covariates (Li & Yu, 2008). These PCs are obtained using multidimensional scaling of the matrix of genetic distances between the isolates. The PCs represent isolates as coordinates in a (virtual) space in which the genetic distances are approximately preserved, such that closely related isolates exhibit similar coordinates. Used as model covariates, these PCs represent the position of each isolate in the population structure and effectively account for its influence on the THD response variable.

## 4 GROUP‐LEVEL CORRELATIONS BETWEEN THD, THE MALTHUSIAN PARAMETER, AND DIVERSITY INDICES

Several aggregate measures can be interpreted as proxies of epidemic success at the level of a group of isolates: (a) the group's prevalence, which reflects possible dominance but provides no information on population dynamics; (b) the genetic diversity of the group, which is comparatively lower in recently expanding lineages; and (c) the Malthusian parameter *r*.

We examined the correlation patterns of THD with these aggregate measures in a well‐documented dataset of *M. tuberculosis* genotypes from a collection of 4,987 isolates of the Beijing lineage (Merker et al., 2015). This lineage is a major driver of the ongoing epidemic of multidrug‐resistant tuberculosis (MDR‐TB) in Eurasia (Merker et al., 2018; Wirth et al., 2008) and Africa. The data include the country of isolation, clonal complex (CC), phenotypic resistance profile, and MDR status. The strain genotypes are 24‐position minisatellite markers (Allix‐Béguec et al., 2014; Supply, Niemann, & Wirth, 2011). The Beijing lineage in Eurasia exhibits a strong population structure. The basal lineage (BL) 7, which is ancestral to other Beijing lineages, is confined to the Korean and Japanese regions. The Beijing lineage has diversified during its geographical spread. CC1 is present in Central Asia, Russia, and Eastern Europe and exhibits ~30% MDR phenotypes. CC2 is mostly found in Russia and Belarus and exhibits the highest MDR prevalence at 75%. CC5 has spread to the Pacific islands and did not develop an MDR phenotype, probably because of specific treatment practices in these countries.

For each CC, we derived the Malthusian parameter *r* from previously established estimates of the current population size *N*_0_, the length of time *t* since the beginning of the last expansion, and the ancestral population size *N*_t_ prior expansion, as *r* = −log(*N*_0_/*N*_t_)/*r* (Merker et al., 2015). The estimates for *N*_0_, *N_t_*, and *t* had been obtained using Bayesian coalescent‐based demographic analyses in the Merker study. Along with the Malthusian parameter, we computed other CC‐level aggregate quantities including prevalence (CC sample size) and three asymptotic measures of diversity, namely richness, the Shannon entropy index, and the Simpson index of diversity (Table 1). Asymptotic estimators (as expected under infinite sample size) of richness, Shannon index, and Simpson index were computed using the package *iNEXT* for the R environment (Hsieh, Ma, & Chao, 2016).

**TABLE 1:** Demographic characteristics of 7 clonal complexes (CC) of *M. tuberculosis* Beijing

CC	Median THD ×100	Malthusian parameter ×1000	*N*	Richness	Shannon index	Simpson index (%)
BL7	5.10	1.5	330	1,012.9	6.4	99.4
CC1	9.56	10.9	892	376.8	2.8	67.9
CC2	9.24	13.0	432	163.5	2.2	55.9
CC3	8.42	6.5	736	790.3	5.3	97.0
CC4	7.44	4.3	732	904.2	5.3	97.8
CC5	7.95	11.2	429	209.0	3.1	82.0
CC6	5.89	2.6	413	1,472.4	6.5	99.3

**FIGURE 2:**
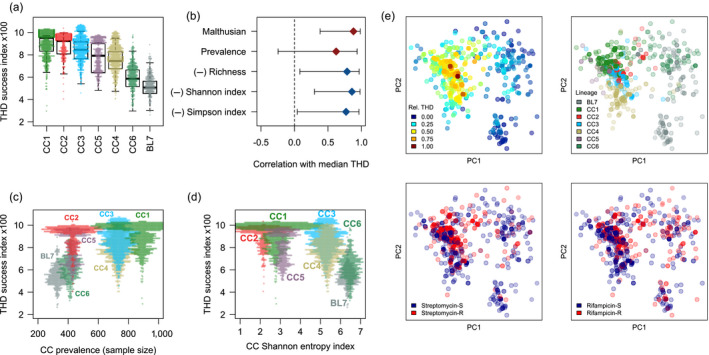
Correlation pattern of mean THD with other aggregated quantities in 7 clonal complexes (CC) of *M. tuberculosis* Beijing. (a) Distribution of THD in CCs. (b) Pearson correlation and 95% confidence interval between the median THD in each CC and other group‐level correlates of epidemic success. Red (blue) markers denote positive (negative) correlation. (c) Distribution of THD relative to the sample size of each CC. (d) Distribution of THD relative to the Shannon entropy index, a measure of diversity. (e) Genetic principal coordinate plots identify partial overlap between higher THD values (upper‐left panel), CC (upper‐right panel), streptomycin resistance (lower‐left panel), and rifampicin resistance (lower‐right panel)

Time‐scaled haplotypic density values were computed from the 24‐MIRU minisatellite data using a per‐marker mutation rate *µ* = 5 × 10^‐4^, a number of markers *m* = 24 as described previously for minisatellites (Rasigade et al., 2017), and a timescale of 200 years consistent with the estimated expansion period of epidemic Beijing sublineages (Merker et al., 2015). The THD distribution identified CC1 and CC2 as the most successful (Figure 2a). THD reflects both the prevalence and homogeneity in the genetic neighborhood of an isolate. To decipher the relationships of THD with other measures of prevalence and clonality, we examined the correlations between the CC‐level average THDs and the aggregated quantities shown in Table 1. The measure that most strongly correlated with THD was the Malthusian parameter, which is also the most direct aggregated correlate of epidemic success (Figure 2b). Among diversity measures, THD exhibited the strongest (negative) correlation with the Shannon entropy index. The weakest correlation was found between THD and prevalence. Details of the distribution of THD relative to CC prevalence and Shannon index are shown in Figure 2c,d, respectively. Collectively, these results indicate that the average THD mostly reflects population expansion in this dataset, as measured through the Malthusian parameter of each CC.

## 5 EPIDEMIC SUCCESS AND ANTIBIOTIC RESISTANCE IN THE *M. TUBERCULOSIS* BEIJING EPIDEMIC

To illustrate the use of an individual‐based correlate of epidemic success, we examined the relationships between drug resistance and success in *M. tuberculosis* Beijing. The wide variations in drug resistance between CCs (Table S1) and regions (Table S2) make it difficult to disentangle the possible effects of each resistance on the success of Beijing lineages. Moreover, the patterns of associations between resistance, CCs and THD were intricate, as illustrated for streptomycin and rifampicin resistance using genetic principal coordinate (PC) visualization (Figure 2e). Therefore, we used mixed‐effects modeling to illustrate how THD analysis can isolate the confounding effect of geographic and population structure from the effect of drug resistance.

Time‐scaled haplotypic density was regressed on resistance to major anti‐TB drugs isoniazid, rifampicin, streptomycin, ethambutol, and pyrazinamide, which were coded as binary predictors. Drug resistances were either included in separate models or together in a multiple regression model (simple versus multiple regression models, respectively). In each case, the model was controlled or not for confounding by geographical and genetic structure by including the CC and the geographic region of the isolate as random effects in a mixed‐effects model (uncontrolled versus controlled models, respectively).

Model coefficients and their significance levels are shown in Table 2. The comparison of bivariate and multiple regression models, along with the comparison between uncontrolled and controlled models, illustrates the patterns of the association between success and resistance. Bivariate models capture all marginal associations between drug resistance and THD, without considering correlation between the resistances. In multiple regression models, the effect of drug resistance is independent of other resistances. Finally, controlled models examine associations independent of the effect of between‐CC and between‐region variations.

**TABLE 2:** Regression models of THD success index on drug resistance profile

Resistance	Bivariate regression				Multiple regression			
	Uncontrolled		Controlled		Uncontrolled		Controlled	
	Coef.	*P*	Coef.	*P*	Coef.	*P*	Coef.	*P*
Isoniazid	10.0	***	2.5	***	0.0	NS	−0.3	NS
Rifampicin	7.7	***	2.8	***	−1.1	NS	1.8	*
Streptomycin	15.3	***	3.8	***	19.7	***	3.5	***
Ethambutol	7.3	***	2.0	***	0.2	NS	0.0	NS
Pyrazinamide	7.1	***	1.9	**	0.9	NS	0.4	NS

Note

Coefficients ×1,000 for readability. Controlled models included the isolate clonal complex and geographic region as random effects.

In bivariate, uncontrolled models, THD was most strongly associated with streptomycin and isoniazid resistance (Table 2), which reflected the comparatively higher rates of resistance to these drugs in the highly successful CC1 and CC2 (Table S1). After controlling for geographic distribution and population structure, rifampicin resistance was the second strongest predictor of THD after streptomycin resistance. In multiple, uncontrolled regression models, streptomycin resistance was the only independent predictor of success, most likely due to its enrichment in CC1 and CC2 (Figure 2e). Interestingly, in the controlled multiple regression model, rifampicin resistance was found as an additional predictor of success.

Overall, the THD analysis suggests that drug resistance, especially to streptomycin and rifampicin, is a driver of transmission success in Beijing lineage isolates. This association was robust to confounding by geographical and genetic structure of the sampled isolates. This indicates that the emergence of drug resistance in the Beijing lineage is not measurably counteracted by a fitness cost, a finding that brings additional support to the need for susceptibility test‐driven anti‐tuberculosis therapy in areas where this lineage is prevalent (Merker et al., 2018).

## 6 CHROMOSOMAL FLUOROQUINOLONE RESISTANCE IN MULTIDRUG‐RESISTANT TYPHOID FEVER

Typhoid caused by *Salmonella* Typhi is endemic in many countries and the emergence of MDR lineages further amplifies its burden. The epidemic of MDR typhoid is mainly driven by the dominant lineage H58. Phylogeographic analysis of H58 and non‐H58 *S*. Typhi isolates has unveiled the historical emergence of the lineage in the Indian subcontinent and its subsequent spread toward Eastern Asia and Africa (Wirth, 2015; Wong et al., 2015). The accumulation of resistance determinants in the 1970s led to the emergence of strains resisting all first‐line drugs including ampicillin, chloramphenicol and trimethoprim‐sulfamethoxazole. This first wave of resistance was followed by the acquisition of chromosomal mutations conferring additional resistance to fluoroquinolones such as ciprofloxacin that have been increasingly used to treat typhoid since the 1990s (Menezes, Harish, Khan, Goessens, & Hays, 2012).

Understanding and quantifying the fitness impact (negative, neutral, or beneficial) of fluoroquinolone resistance in *S. *Typhi is critical for informing antimicrobial chemotherapy policy and anticipating the evolution of resistance. Chromosomal resistance to fluoroquinolones typically involves mutations in the genes encoding the DNA gyrase (*gyr*A, *gyr*B) and topoisomerase IV (*par*C or *par*E). Such mutations in essential, housekeeping genes have been repeatedly associated with a strong fitness cost in pathogenic bacteria including non‐Typhi *Salmonella enterica* (Giraud, Cloeckaert, Baucheron, Mouline, & Chaslus‐Dancla, 2003; O’Regan et al., 2010). However, this fitness cost was less apparent in fluoroquinolone‐resistant *S*. Typhi. In in vitro competition experiments, several fluoroquinolone‐resistant (FQ‐R) *S. *Typhi mutants outcompeted their susceptible progenitors in FQ‐free medium, suggesting that FQ‐R associated mutations do not necessarily entail a fitness cost and might even benefit their carrier in the absence of antibiotic pressure (Baker et al., 2013). Yet, our knowledge of the impact of fluoroquinolone resistance on fitness derives from laboratory experiments. This impact has not yet been examined in an epidemiological setting.

In this context, we illustrate the use of THD to address the question of whether fluoroquinolone resistance has a measurable impact on pathogen success at population scale. We used a genomic dataset generated from an international study of H58 and non‐H58 *S. *Typhi (Wong et al., 2015). The dataset is comprised of 1,832 whole‐genome sequences of isolates from 68 countries, mainly in Asia and Africa. The genomic correlate of chromosomal fluoroquinolone resistance was the presence of FQ‐R‐associated mutations *gyr*A, *gyr*B, *par*C, or *par*E. Of note, non‐chromosomal fluoroquinolone resistance conferred by plasmid‐borne *qnr* genes was exceptional in the collection (*n* = 7). THD estimates were computed from the matrix of pairwise SNP distances using a timescale of 20y, an effective genome size of 4.6 × 10^6^ bp and an average per‐nucleotide substitution rate of 1.4 × 10^−7^ year^−1^ as estimated previously using Bayesian coalescent‐based analysis (Wong et al., 2015).

**FIGURE 3:**
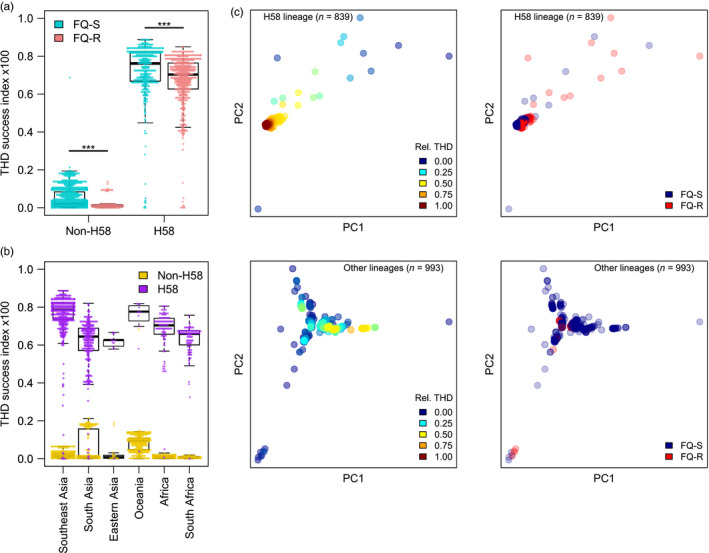
Distribution of the THD success index in *Salmonella* Typhi H58 and non‐H58 isolates depending on chromosomal fluoroquinolone resistance (a) and geographic origin (b). (c) Genetic principal coordinate plots illustrate lack of overlap between higher THD values (left column) and fluoroquinolone resistance (right column) both in H58 and non‐H58 lineages (upper and lower rows, respectively). ***p < .001, Mann–Whitney *U* test

The THD index was >10‐fold higher in FQ‐R isolates compared to FQ‐S isolates (median 6.8 × 10^−3^ versus 5.4 × 10^−4^, *p* < .0001, Mann–Whitney *U* test). Fluoroquinolone resistance, however, was strongly associated with the H58 haplotype (odds ratio 14.0, 95% CI 10.8 – 18.2) that also exhibited >30‐fold higher median THD (7.2 × 10^−3^ versus 1.9 × 10^−4^). Thus, the positive relationship between THD and fluoroquinolone resistance was strongly driven by FQ‐R enrichment within H58 isolates. Strikingly, the same analysis conducted separately on H58 and non‐H58 groups (*n* = 839 and 993, respectively) revealed a negative, significant association between fluoroquinolone resistance and THD in both groups (Figure 3a,c).

This pattern is compatible with the hypothesis that, at the population scale, chromosomal fluoroquinolone resistance entails a measurable fitness cost in both H58 and non‐H58 isolates, but that widespread fluoroquinolone use sustains a high prevalence of resistance. To challenge this hypothesis while illustrating the use of THD in more complex applications, we constructed additional models controlling for population structure and geographic distribution. In our setting, the geographic origin can capture potential differences in fluoroquinolone use between regions, which might also correlate with the distribution of fluoroquinolone resistance, H58 prevalence and its transmission success (Figure 3b). To control for confounding by geographic origin, isolates were assigned to 6 regions whose areas were arbitrarily defined to avoid sample sizes <30. Details of the distribution of isolates in countries and regions can be found in the Table S3. The region of origin was included as a random effect in the controlled model.

Controlling for population structure is especially important in this application because THD depends on population structure by design and FQ‐R mutations are vertically inherited. Contrasting with the previous application on *M. tuberculosis* Beijing, in which an appropriate number (*n* = 7) of well‐defined clonal complexes with balanced sample sizes were available as a control variable, the *S. *Typhi collection is comprised of the dominant and highly clonal H58 lineage and a more diverse background of other lineages. In this context, including the H58 genetic background as a binary control variable is insufficient because the population structure within H58 and other lineages is not accounted for. To circumvent this issue without defining arbitrary lineages or clades, the model was adjusted for population structure using genetic PCs computed from the SNP distance matrix (Li & Yu, 2008; Price et al., 2006). PCs (*n* = 30) were first included in a linear mixed‐effect model regressing THD on the presence of chromosomal fluoroquinolone resistance, a H58 genetic background and, as a random effect, the region of origin. This full model was used to select relevant PCs, defined as PCs with a coefficient *p*‐value <.01. The selected PCs (*n* = 14) were included in a new model, keeping other predictors unchanged. In this final model, chromosomal fluoroquinolone resistance retained a negative association with the THD success index, with a coefficient of −2.5 × 10^−4^ (95% CI, −3.7 × 10^−4^ to −1.2 × 10^−4^, *p* < .0001). Collectively, these results support the existence of a moderate but measurable fitness cost of chromosomal fluoroquinolone resistance at population level in *S. *Typhi, independent of confounding by population structure and geographic origin.

## 7 DRIVERS OF EPIDEMIC SUCCESS IN COMMUNITY‐ACQUIRED METHICILLIN‐RESISTANT *S. AUREUS*

In the late 1990s and early 2000s, an epidemic of methicillin‐resistant *S. aureus* (MRSA) stormed North America, driven by a highly clonal, dominant lineage, USA300, that belongs to the multilocus sequence type (ST) 8 (Diep et al., 2006). The USA300 lineage is thought to have diverged from ancestral methicillin‐susceptible strains harboring the phage‐borne genes *luk*S‐PV and *luk*F‐PV encoding the Panton‐Valentine leukocidin (PVL), a potent pro‐inflammatory toxin. The acquisition of the drug resistance cassette SCC*mec* led to the emergence of the MRSA USA300 lineage, which has then evolved into two separate clades: USA300‐NA (North America), mainly distributed in the United States, characterized by the presence of the arginine catabolic mobile element (ACME) type I; and USA300‐LV (Latin Variant), distributed in Colombia, Ecuador, and Venezuela, lacking ACME. The ACME element, whose presence enhanced the virulence and persistence of *S. aureus* in animal models of skin infection (Thurlow et al., 2013), may have contributed to the successful spread of the USA300‐NA variant. A few years after their emergence, USA300 isolates evolved chromosomal mutations conferring resistance to fluoroquinolones. In Bayesian coalescent‐based demographic analyses, the successive acquisition of ACME and fluoroquinolone resistance coincided with two phases of expansion of the effective population size of USA300 (Glaser et al., 2016). These findings suggested that both acquisition events contributed additively to the success of USA300‐NA. However, the associations of ACME and fluoroquinolone resistance acquisition with population expansion phases were coincidental in nature and do not provide a definitive evidence of a role of these traits in the epidemic success of USA300 relative to its ST8 siblings.

Interestingly, the role of fluoroquinolone resistance in the success of MRSA is supported by several lines of evidence. Fluoroquinolone resistance, which has emerged repeatedly in the history of USA300 (Glaser et al., 2016), has also been associated with the expansion of other MRSA lineages in other geographic regions (McAdam et al., 2012). Moreover, limiting prescription of fluoroquinolones was shown in independent studies to correlate with a decrease in MRSA prevalence (Charbonneau et al., 2006; Parienti et al., 2011). Finally, fluoroquinolone concentrations as small as 1/100 of the wild‐type minimum inhibitory concentration allowed a FQ‐R variant to outcompete its susceptible progenitor in co‐culture experiments (Gustave et al., 2018).

Compared with fluoroquinolone resistance, the role of ACME in the success of USA300 is less supported. The role of ACME in the virulence of infection was not consistent in animal models (Montgomery, Boyle‐Vavra, & Daum, 2009), and an epidemiological link of ACME with more severe disease in humans is still lacking. ACME acquisition coincided with the emergence of USA300‐NA, but this acquisition was a unique event and ACME is very seldom found in other *S. aureus* lineages. Finally, phylogenetic analyses have shown that ACME was lost on several occasions by USA300 isolates (Jamrozy et al., 2016).

In this context, we leveraged the THD framework to examine the relative contributions of ACME and fluoroquinolone resistance to the epidemic success of USA300 and other representatives of the ST8 lineage. We used the genome sequences of 498 ST8 isolates from the United States and France that were previously analyzed by our group to examine the emergence of USA300 from the ST8 genetic background (Glaser et al., 2016). Chromosomal fluoroquinolone resistance was inferred based on mutations in *gyr*A and *par*C, and the presence of ACME was determined based on sequence alignment. THD estimates were computed from core‐genome SNP distances using a timescale of 20y, an effective genome size of 2.7 × 10^6^ bp, and an average per‐nucleotide substitution rate of 1.3 × 10^−6^ year^−1^ as determined previously using Bayesian coalescent‐based analysis (Glaser et al., 2016).

**FIGURE 4:**
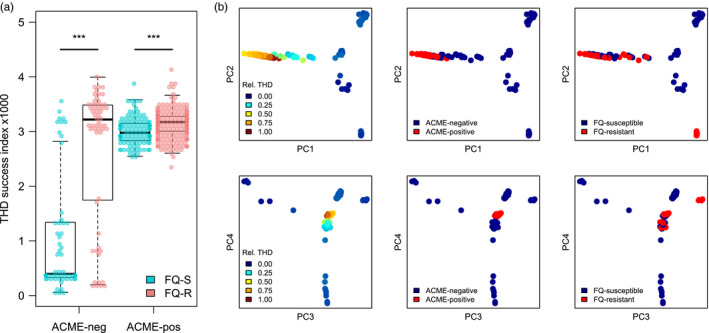
Distribution of THD success index in *S. aureus* ST8 isolates depending on the presence of ACME and of chromosomal fluoroquinolone resistance (FQ‐R). (a), fluoroquinolone resistance is associated with increased THD values in ACME‐negative and ‐positive isolates. (b) genetic principal coordinate plots of ST8 isolates in the two first PC planes identifies partial overlap between higher THD values (left column), ACME positivity (center column) and fluoroquinolone resistance (right column). ***p < .001, Mann–Whitney *U* test

The median THD value was 1.6× higher in the 385 (77.3%) ACME‐positive ST8 isolates compared to the 113 (22.7%) other ST8 isolates (2.7 × 10^−3^ versus 1.5 × 10^−3^, respectively, *p* < 10^–6^, Mann–Whitney *U* test; Figure 4a). Interestingly, the median THD was very close in the 322 (64.7%) FQ‐R and the 176 (35.3%) FQ‐S isolates (2.8 × 10^−3^ versus 2.6 × 10^−3^, respectively); however, the THD distribution in FQ‐R isolates was stochastically greater (*p* < 10^–16^). In separate subgroup analyses of ACME‐positive and ACME‐negative isolates, the THD distribution was stochastically greater in the FQ‐R members of both groups (Figure 4a; *p* = 3.3 × 10^−8^ in ACME‐positive and *p* = 2.2 × 10^−8^ in ACME‐negative isolates). Using similar subgroup analyses in FQ‐R and FQ‐S isolates, the THD distribution of ACME‐positive isolates was stochastically greater in the FQ‐S group (*p* = 6.1 × 10^−6^) but not in the FQ‐R group (*p* =.46), suggesting that ACME positivity does not add to the success of FQ‐R isolates.

Fluoroquinolone resistance was strongly associated with the presence of ACME (odds ratio 2.27 [95%CI, 1.45 to 3.57], *p*=.0002, Fisher's exact test). Thus, as in the case of *S*.Typhi H58 discussed above, the predictors of interest (here, ACME and fluoroquinolone resistance) correlated both with THD and with each other, as illustrated by the overlap of ACME‐positive and FQ‐R isolates in genetic PC visualization (Figure4b). To disentangle this tight correlation structure, we used multiple regression controlling for geographic distribution and population structure. Noteworthy, our ST8 collection was dominated by US isolates (*n*=431, 86.5%). Geographic origin did not correlate with either THD (*p*=.48, Mann–Whitney *U* test) or with fluoroquinolone resistance (*p*=.13, Fisher's exact test), but all ACME‐negative isolates were collected from the United States, which motivated the inclusion of a US origin as a control covariate. Because only two geographic origins were present, we used a fixed effect rather than a random effect. Using a similar model‐building procedure as described for *S*.Typhi, we first identified the relevant genetic PCs predicting THD in a complete model (*n*=7 PCs with *p*<.01). In the final, controlled model with 7 PCs, fluoroquinolone resistance was predictive of higher THD values, with a coefficient of 6.1×10^−5^ (95% CI, 4.4×10^−6^ to 1.2×10^−4^, *p*=.03). The presence of ACME, however, did not predict THD (*p*=.25) and, of note, had a negative coefficient. Collectively, these findings provide additional support for the hypothesis that fluoroquinolone resistance drives the success of MRSA lineages including USA300. We failed, however, to identify an independent contribution of ACME to the success of USA300.

## 8 CONCLUSIONS

Our main goal here was to present applications of a fast and efficient technique to estimate the epidemic success of pathogens. As illustrated in the previous sections, the THD method provides researchers with a flexible index of epidemic success, which can be used in regression analysis in the same manner as a quantitative trait. Combined with multivariate modeling, this approach revealed contrasting behaviors in different bacterial species and lineages, such as a potential benefit of fluoroquinolone resistance in *S. aureus* ST8 but not *S*. Typhi H58. More generally, our findings suggest that some lineages develop antibiotic resistance without any detectable fitness costs, as exemplified by the Beijing lineage of *M. tuberculosis*, whereas other major outbreaks like the *Salmonella* Typhi H58 lineage pay a price for resistance.

The reasons behind these contrasting results are still elusive, but our analyses strengthen our understanding of the worldwide success of the Beijing *M. tuberculosis* lineage (Barbier & Wirth, 2016). Lack of detectable fitness cost in MDR strains could result from compensatory evolution, a famous example being *rpo*B compensatory mutations in *M. tuberculosis*. Yet, other mechanisms accompanying MDR clades could hide the fitness cost of resistance. For instance, putative counterbalancing apparatus might accelerate the generation time or mutation rate, indirectly improving transmission and fitness (Ford et al., 2013).

To resolve these questions with greater accuracy, density‐based approaches such as THD bear the potential for being implemented in genome wide association studies (GWAS) (Chen & Shapiro, 2015; Jaillard et al., 2018; Power, Parkhill, & de Oliveira, 2017). Treated as a quantitative trait, THD might be associated with specific SNPs or with more complex underlying genetic architectures, as exemplified in a recent study of the impact of vancomycin resistance‐associated SNPs on the success of the opportunistic pathogen *Staphylococcus capitis* (Wirth et al. 2020). The widespread adoption of THD‐based GWAS might help to widen the spectrum of the candidate genes or mutations linked to pathogen success, thus accompanying a shift from the current, paradigmatic antibiotic resistance‐targeted GWAS approaches to more general questions pertaining to pathogen ecology and evolution (Farhat et al., 2019; Hicks, Carey, Yang, Zhao, & Fortune, 2019).

## CONFLICT OF INTEREST

None declared.

## Supporting information

Supplementary Material

## Data Availability

The data that support the findings of this study are available from the corresponding author upon reasonable request.
