# Supplementary material for: Applied phyloepidemiology: Detecting drivers of pathogen transmission from genomic signatures using density measures
Source: Evol Appl. 2020 May 22;13(6):1513–25. doi: 10.1111/eva.12991 (PMC7359849; doi:10.1111/eva.12991)
Supplement: Supplementary file 1 — Supplementary Material [file EVA-13-1513-s001.docx]

| **Supplementary Table 1. Proportion of resistance to anti-TB drugs in 7 clonal complexes of the Beijing lineage.** | | | | | | | |
| --- | --- | --- | --- | --- | --- | --- | --- |
|  | **% resistance** | | | | | | |
| **Resistance** | **BL7** | **CC1** | **CC2** | **CC3** | **CC4** | **CC5** | **CC6** |
| Isoniazid | 41.1 | 74.3 | 92.7 | 40.6 | 29.9 | 11.7 | 48.6 |
| Rifampicin | 41.1 | 60.9 | 85.0 | 32.9 | 21.9 | 8.3 | 48.6 |
| Streptomycin | 22.3 | 77.5 | 95.1 | 40.2 | 29.3 | 7.5 | 26.1 |
| Ethambutol | 28.6 | 52.2 | 59.4 | 19.9 | 14.9 | 4.4 | 28.6 |
| Pirazinamide | 19.2 | 41.6 | 51.1 | 18.9 | 15.8 | 7.7 | 28.6 |

| **Supplementary Table 2. Proportion of resistance to anti-TB drugs in Beijing lineage isolates from 7 geographic regions.** | | | | | | | |
| --- | --- | --- | --- | --- | --- | --- | --- |
| **Resistance** | **Europe** | **Russia** | **Central Asia** | **Eastern Asia** | **Southern Asia** | **Pacific** | **Africa** |
| Isoniazid | 71.8 | 90.6 | 79.3 | 40.2 | 14.3 | 13.6 | 23.5 |
| Rifampicin | 62.2 | 81.2 | 64.6 | 41.3 | 8.6 | 5.1 | 20.0 |
| Streptomycin | 76.1 | 91.8 | 81.8 | 21.8 | 23.1 | 13.3 | 12.1 |
| Ethambutol | 44.3 | 75.0 | 54.6 | 26.5 | 11.4 | 5.1 | 9.6 |
| Pirazinamide | 47.9 | 46.9 | 40.2 | 17.9 | 5.7 | 4.2 | 0.0 |

| **Supplementary Table 3. Geographical distribution of 1,873 *S.* Typhi isolates.** | | |
| --- | --- | --- |
| **Country** | **Region** | **No. of isolates** |
| Iraq | Eastern Asia | 11 |
| Iran | Eastern Asia | 3 |
| Russia | Eastern Asia | 1 |
| Lebanon | Eastern Asia | 7 |
| Armenia | Eastern Asia | 1 |
| Turkey | Eastern Asia | 2 |
| Palestine | Eastern Asia | 1 |
| Kuwait | Eastern Asia | 2 |
| Bangladesh | South Asia | 90 |
| India | South Asia | 194 |
| Pakistan | South Asia | 57 |
| Nepal | South Asia | 48 |
| Sri Lanka | South Asia | 3 |
| Afghanistan | South Asia | 1 |
| Indonesia | Southeast Asia | 126 |
| Cambodia | Southeast Asia | 210 |
| Laos | Southeast Asia | 138 |
| Thailand | Southeast Asia | 7 |
| Philippines | Southeast Asia | 3 |
| Vietnam | Southeast Asia | 218 |
| Myanmar | Southeast Asia | 4 |
| Malaysia | Southeast Asia | 2 |
| East Timor | Southeast Asia | 1 |
| Fiji | Oceania | 170 |
| Samoa | Oceania | 117 |
| Tonga | Oceania | 3 |
| Papua New Guinea | Oceania | 45 |
| Australia | Oceania | 3 |
| Vanuatu | Oceania | 2 |
| Tanzania | Africa | 52 |
| Kenya | Africa | 55 |
| Democratic Republic of the Congo | Africa | 17 |
| Liberia | Africa | 1 |
| Central African Republic | Africa | 6 |
| Sudan | Africa | 2 |
| Egypt | Africa | 3 |
| Algeria | Africa | 7 |
| Morocco | Africa | 8 |
| Ivory Coast | Africa | 4 |
| Senegal | Africa | 2 |
| Gabon | Africa | 1 |
| Comoros | Africa | 3 |
| Cameroon | Africa | 27 |
| Guinea | Africa | 2 |
| Madagascar | Africa | 4 |
| Mali | Africa | 4 |
| Nigeria | Africa | 3 |
| Mauritania | Africa | 2 |
| Burkina Faso | Africa | 3 |
| Niger | Africa | 1 |
| Tunisia | Africa | 3 |
| Benin | Africa | 4 |
| Angola | Africa | 2 |
| Cape Verde | Africa | 1 |
| Togo | Africa | 3 |
| Ghana | Africa | 1 |
| South Africa | South Africa | 41 |
| Malawi | South Africa | 112 |
| China | -* | 3 |
| Paraguay | -* | 2 |
| Mexico | -* | 3 |
| Malta | -* | 1 |
| El Salvador | -* | 1 |
| Argentina | -* | 10 |
| Peru | -* | 2 |
| France | -* | 4 |
| French Guiana | -* | 2 |
| United States | -* | 1 |
| NOTE. *, countries were excluded from region-based analysis due to small sample size in the resulting region. | | |


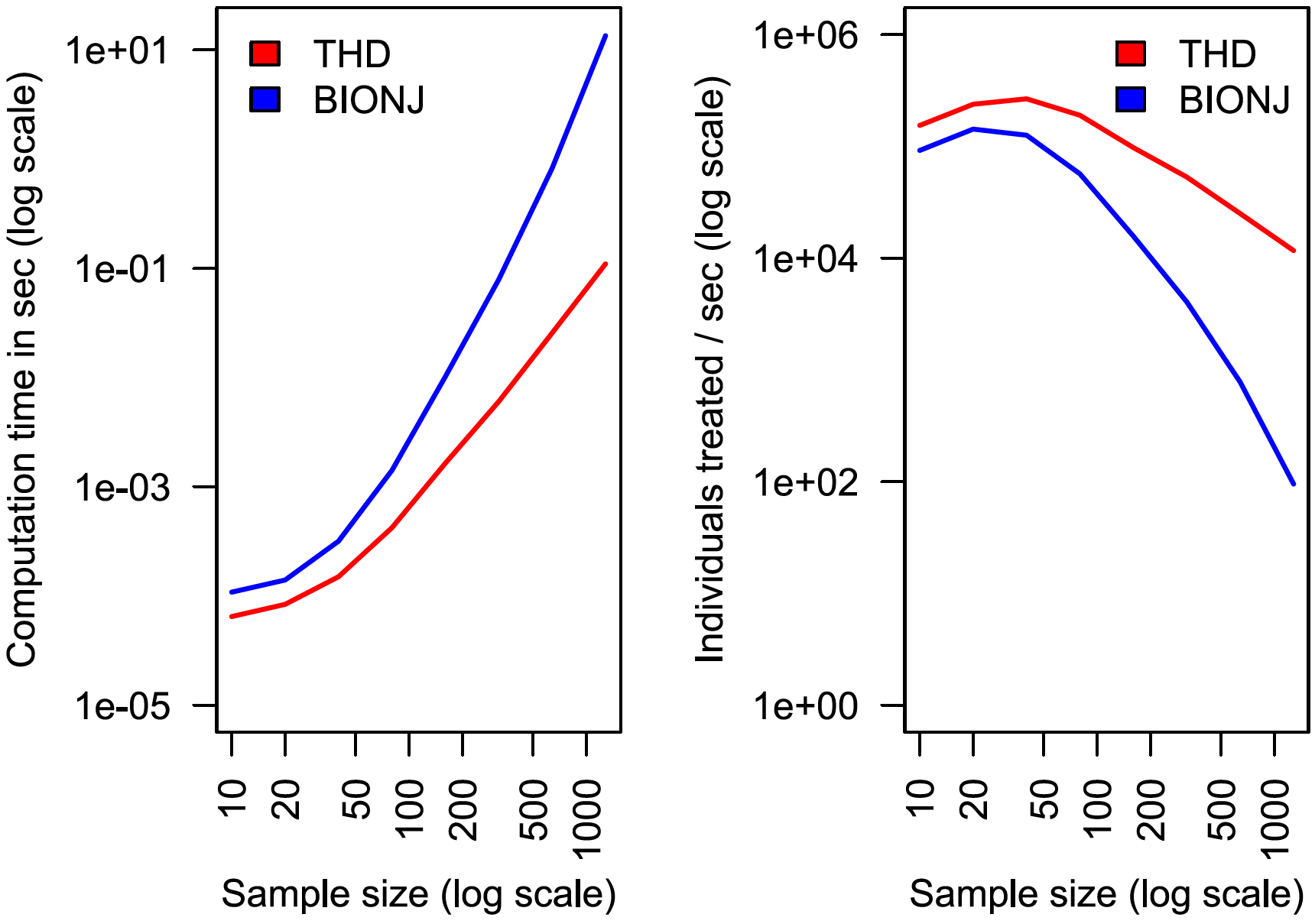


**Supplementary Figure 1. THD computation time benchmark and comparison with the phylogeny reconstruction method BIONJ.** Genetic distance matrices of increasing sizes were used as input to the THD computation routine in R package *thd* and, for comparison, to the fast phylogeny reconstruction method BIONJ as implemented in R package *ape*. Shown are the median computation time (left panel) and the no. of individuals treated per second (right panel) in 10 replicates. Interquartile ranges are within the line width for both methods.
